# Retraction: In-depth systems biological evaluation of bovine alveolar macrophages suggests novel insights into molecular mechanisms underlying *Mycobacterium bovis* infection

**DOI:** 10.3389/fmicb.2025.1605607

**Published:** 2025-04-10

**Authors:** Frontiers Editorial Office

The Journal retracts the 2022 article cited above.

Following publication, concerns were raised regarding the contributions and affiliations of the authors, as well as the validity of the qRT-PCR data presented in the article. The authors failed to provide a satisfactory explanation during the investigation, which was conducted in accordance with Frontiers' policies. Given the concerns, the editors no longer have confidence in the findings presented in the article.

This retraction was approved by the Chief Executive Editor of Frontiers. The authors received a communication regarding the retraction and had a chance to respond. This communication is recorded by the publisher.

